# Complete Nucleotide Sequence of pVv01, a P1-Like Plasmid Prophage of *Vibrio vulnificus*

**DOI:** 10.1128/genomeA.00135-14

**Published:** 2014-08-14

**Authors:** Jens A. Hammerl, Karina Klevanskaa, Eckhard Strauch, Stefan Hertwig

**Affiliations:** Federal Institute for Risk Assessment (Bundesinstitut für Risikobewertung), Berlin, Germany

## Abstract

We report here the 79,263-bp plasmid pVv01 isolated from *Vibrio vulnificus*. pVv01 is closely related to the *Vibrio* plasmid p0908 and shows some similarities to phage P1. Unlike p0908, pVv01 represents an intact prophage inducible by mitomycin C. PVv01 phage particles revealed a myoviridal morphology and lytic activity.

## GENOME ANNOUNCEMENT

*Vibrio vulnificus* is an important pathogen that can cause serious infections in humans (1, 2). Yet, there is limited knowledge of its virulence factors and of plasmids and phages that might play a role in its pathogenicity.

Here, we report the complete nucleotide sequence of plasmid pVv01 of the *V. vulnificus* biovar 1 strain VN-0101 isolated from the Baltic Sea (3). The plasmid was isolated using the alkaline extraction procedure (4), followed by buoyant density ultracentrifugation (5). Whole-genome sequencing was performed with the Roche 454 genome sequencer GS-FLX Titanium system (LGC Genomics, Germany). Assembly of the reads (Newbler Assembler 2.8) yielded a single linear contig with an 18-fold sequence coverage. Gene prediction and annotation of the genome sequence were carried out using MyRAST (6–8).

Plasmid pVv01 is a circular double-stranded DNA of 79,263 bp, with an average G+C content of 46.4%. One hundred nine putative genes and 10 rho-independent transcription terminators have been identified. For 97 of the predicted products, similarities to known proteins were found, 84 of which are related to proteins of the *Vibrio* plasmid p0908 (accession no. NC_010113.1) (9), *Vibrio owensii* strain LMG 25443 (BioProject no. PRJDB728), *Alteromonas macleodii* plasmid pAMBAS45 (accession no. NC_018680.1), and enterobacterial phage P1 (accession no. NC_005856.1). Moreover, the pVv01 sequence revealed a similar arrangement and orientation of the genes as in p0908 (9) and P1 (10). Notably, genes that probably encode replication and recombination proteins, as well as phage-related proteins, are clustered in pVv01. In p0908, isolated from an environmental *Vibrio* strain, 23 assigned products are similar to those of phage P1 proteins, but the plasmid probably lacks some genes essential for phage propagation (9). Plasmid pVv01 contains 26 genes whose products are related to P1 proteins, with identity values between 24 and 41%. We found products for virion structure (head, tail, and baseplate), replication, plasmid partitioning (ParA) and recombination (Cre), antirestriction (DarA and DarB), DNA packaging (PacB), and host cell lysis. As with p0908, genes for the pacase subunit PacA and a holin were not identified. However, 13 pVv01 products not related to P1 proteins showed some similarities to those of other phages.

To elucidate whether pVv01 is an inducible prophage, an induction experiment with mitomycin C was carried out (11). Lysates examined by electron microscopy displayed particles with an icosahedral head and a long contractile tail typical of the family *Myoviridae*. The phage DNA showed nearly identical restriction patterns as those of the pVv01 plasmid, with one additional band in some digests. Bal31 analyses revealed that the phage genome is a linear circular permuted molecule. Whether it is terminally redundant, like the P1 genome, has still to be unraveled. We performed plaque assays with 20 *V. vulnificus* strains and found that the phage formed turbid plaques on strain VN-0260 (3). Thus, unlike p0908, plasmid pVv01 represents an intact prophage that shows some relationship to enterobacterial phage P1. To our knowledge, this is the first report of an active P1-like phage isolated from a marine bacterium.

### Nucleotide sequence accession number.

The nucleotide sequence of plasmid pVv01 is available under the Genbank accession no. HG803186.
